# Spectroscopic secondary structure fingerprint of β-variant of SARS-CoV-2 spike glycoprotein

**DOI:** 10.1007/s00249-025-01782-8

**Published:** 2025-07-21

**Authors:** Rosanna Mosetti, Tiziana Mancini, Federica Bertelà, Salvatore Macis, Nicole Luchetti, Velia Minicozzi, Stefano Lupi, Annalisa D’Arco

**Affiliations:** 1 Department of Basic and Applied Sciences for Engineering (SBAI), Sapienza University of Rome, Via A. Scarpa 16, 00161 Rome, Italy; 2 Department of Physics, Sapienza University of Rome, P.le A. Moro 2, 00185 Rome, Italy; 3 Engineering Department, Università Campus Bio-Medico di Roma, Via Alvaro del Portillo 21, 00128 Rome, Italy; 4 Centre for Life Nano- and Neuro-Science, Italian Institute of Technology, Viale Regina Elena 291, 00161 Rome, Italy; 5 Department of Physics and INFN, University of Rome Tor Vergata, Via della Ricerca Scientifica, 1, 00133 Rome, Italy

**Keywords:** ATR-IR spectroscopy, Protein secondary structure, Hydrophilicity, DSSP assignment, Molecular dynamics, SARS-CoV-2 spike glycoprotein, Variants

## Abstract

The global outbreak of COVID-19 pandemic has been accompanied by the emergence of numerous mutated forms of the SARS-CoV-2 virus, exhibiting an increasingly refined capacity to adapt to the human host. The majority of mutations affect viral proteins, particularly the Spike glycoprotein (S), leading to alterations in their physicochemical properties, in secondary structures and biological functions. In the present work, we performed, to the best of our knowledge, the first infrared spectroscopic characterization of monomeric spike glycoprotein subunits 1 (S1) of SARS-CoV-2 Beta variant at pH 7.4, combining the experimental results with Molecular Dynamic simulations, Definition of Secondary Structure of Proteins (DSSP) assignments and hydrophobicity calculations. This integrated approach has yielded valuable insights into the protein secondary structure, hydrophobic behaviour, conformational dynamics, and functional attributes, factors essential for a comprehensive understanding of the viral protein domain. Our results reveal that the SARS-CoV-2 S1 Beta variant is characterized by a secondary structure enriched with antiparallel β-sheets, as consistently supported by both experimental data and computational models. Moreover, a comparative analysis of the experimental results with hydrophobicity calculations indicates that the Beta variant exhibits a slightly more hydrophilic nature relative to the SARS-CoV-2 S1 Wild Type.

**Supplementary Information:**

The online version contains supplementary material available at 10.1007/s00249-025-01782-8.

## Introduction

Since 2019, a new virus from the beta Coronavirus family, named SARS-CoV-2, has been circulating, causing the largest-scale health emergency in recent centuries, with more than 7 million deaths to date, as declared by the World Health Organization (WHO) (World Health Organization Data 2025). Morphologically, SARS-CoV-2 exhibits a structure similar to that of other Coronaviruses, characterized by a spherical enveloped structure (with a diameter between 80 and 120 nm) containing a single positive-strand RNA. The viral envelope is decorated with several proteins, such as envelope (E), membrane (M), nucleocapsid (N), spike (S) and other lipid-associated proteins (VerMa and Subbarao 2021; Li et al. 2020; Mancini et al. 2024). Among them, the spike (S) glycoprotein is the largest viral protein, consisting of 1273 amino acids. It protrudes from the viral surface envelope and is organized in trimers. S protein plays a key role in the initial stages of viral transmission. It is composed of two subunits, S1 (residues 1–685) and S2 (residues 686–1273), each comprising multiple domains. S1 subunit includes the N-terminal domain (NTD, residues 14–305) and the receptor binding domain (RBD, residues 319–541), the latter of which is responsible for binding to the host cell receptor ACE2, thereby initiating the infectious process (VerMa and Subbarao 2021; Li et al. 2020; Mancini et al. 2024; D’Arco et al. 2023). Both subunits, S1 and S2, emerged as key targets for vaccine development, with a range of immunization strategies designed to stimulate neutralizing antibodies and cellular immune responses against these antigenic determinants (Krammer 2020).

**Fig. 1:**
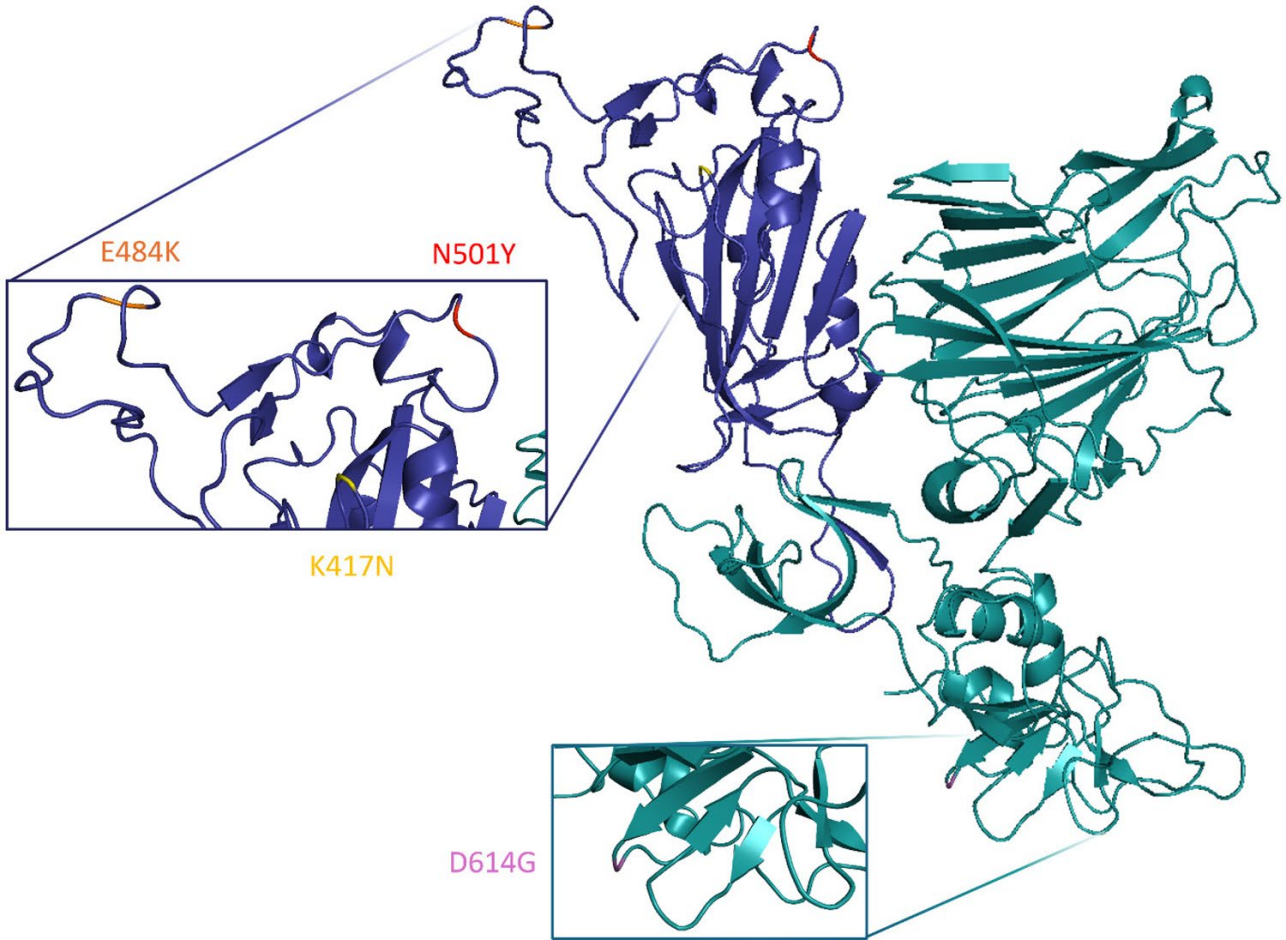
3D Visualization of the monomeric SARS-CoV-2 S1 glycoprotein Beta variant. The RBD is shown in a darker colour and mutations compared to the SARS-CoV-2 S1 wild type are zoomed in and highlighted with different colours: K417N in yellow, E484K in orange, N501Y in red and D614G in pink. PyMOL was used for visualization

The rapid circulation of the virus among human hosts has given rise to new mutant forms known as variants of concern (VoCs). Compared to the progenitor virus, VoCs exhibit greater transmissibility and the ability to increase disease severity. One of the first VoCs to emerge globally was the Beta variant, first observed in a metropolitan area of South Africa at the beginning of December 2020 (World Health Organization Data 2025; Tang et al. 2021). This mutated form spread rapidly and became dominant across all South Africa provinces within a few weeks, eventually being exported to most parts of the world (World Health Organization Data 2025; Tang et al. 2021; Choi and Smith 2021; Tegally et al. 2021). Experimental studies on this lineage indicate that it is more resistant to vaccines compared to the Wild Type (WT) and other VoCs, showing the greatest reduction in neutralizability by sera from vaccinated individuals or convalescent patients (Tegally et al. 2021; Corbett et al. 1979). The decreased susceptibility to vaccine-induced and infection-induced immune responses enhances the variant’s ability to reinfect individuals who were previously infected and had recovered, facilitating large-scale transmission. This acquired ability is associated with a specific Beta variant. SARS-CoV-2 S of Beta variant includes nine mutations (L18F, D80A, D215G, R246I, K417N, E484K, N501Y, D614G, and A701V), respecting the WT virus. Among these, four mutations are in the S1 subunit (K417N, E484K, N501Y and D614G), and three of them are in the RBD domain (K417N, E484K, and N501Y), see Fig. 1.

The mutations present in the RBD region (K417N, E484K and N501Y) are associated with enhanced binding affinity to human ACE2 and increased resistance to vaccine-induced immunity (Choi and Smith 2021; RaManathan et al. 2021; Khan et al. 2021; Starr et al. 2020; Davies et al. 2021). In particular, the N501Y mutation is present in many VoCs, including Alpha, Gamma and Omicron variants, and contributes to increased S protein binding affinity. Meanwhile, the E484K mutation, first identified in the Beta variant, is associated with reduced neutralization by vaccine–boosted (Choi and Smith 2021; Corbett et al. 1979; Jangra et al. 2021). To the best of our knowledge, our study presents the first comparative analysis of monomeric S1 of the Beta variant of SARS-CoV-2 virus, using both experimental and computational approaches. We performed infrared (IR) spectroscopy, a well-established experimental method for a non-invasive and non-destructive analysis of polypeptides and proteins (Mancini et al. 2024; D’Arco et al. 2023; Yang et al. 2015; Barth 2007, 2000; Santos et al. 2021), in combination with Molecular Dynamics (MD) simulations and Definition of Secondary Structure of Proteins (DSSP) assignments. An in-depth examination of the secondary structure was carried out, focusing on the amide I band absorption (1590–1710 cm^−1^) and analyzing its vibrational spectral components (Yang et al. 2015; Barth 2007, 2000; Kong and Yu 2007). IR spectra are significantly influenced by the overall three-dimensional (3D) conformation of the protein, including its intrinsic conformational order in terms of bond and angle symmetry (Barth 2000; Moran and Zanni 2014; Vosough and Barth 2021; Woutersen and Hamm 2001), as well as its folding, packaging, hydrophilicity, and the strength of hydrogen bonds with surrounding water (Myshakina et al. 2008; Manas et al. 2000). Spectral differences observed in the S1 of the Beta variant, compared to S1 WT (Mancini et al. 2024; D’Arco et al. 2023), were further analyzed using computational approaches to investigate its behaviour in an aqueous environment. These approaches included structure prediction via ColabFOLD, MD simulations, and a hydrophobic surface analysis through Protein-sol software. Our results offer valuable insights into the secondary structure and conformational dynamics of the protein in water, thereby enhancing the understanding of its functional properties and the structural dynamics that drive the infectious mechanism. These findings provide a foundation for a range of applications, including the development of innovative optical biosensors, where viral proteins may serve as potential biomarkers (KiTane et al. 2021; Lee et al. 2024; KuMar et al. 2022; Tan et al. 2024; Funari et al. 2020; GaMage et al. 2022), as well as drug design, preventive strategies, and further structural investigations.

## Materials and methods

### Protein preparation

A truly monomeric, non-trimerizing form of the S1 subunit of SARS-CoV-2 Beta variant S glycoprotein was considered. Lineage B. 1.151/B.1351.2/B.1351.3 (Cat No. 40591-V08H10, aa 681, purity > 90%) was purchased from Sino Biological Europe GmbH (Eschborn, Germany). The protein was expressed in baculovirus insect cells with purity > 90% (determined by sodium dodecyl sulphate–polyacrylamide gel electrophoresis SDS-PAGE) and used without further purification. It differs from the S1 protein of SARS-CoV-2 wild type (WT, the variant that affected Wuhan in 2019) (Cat. No. 40591-V08B1, aa 681, purity > 90%) for a number 4 of mutations. Its amino acid sequence is reported in SI (paragraph S1), and its mutations are listed in Table 1.

**Table 1:** Beta Variant of Concern (VoCs), object of our work, and its mutations with respect to the Wuhan WT S1 protein domain (Cat. No. 40591-V08B1). Legend of mutations acronyms: A(000)B means that amino acid A in position (000) has been substituted with amino acid B

Variant	Lineage	Mutations
H10 Beta β	B. 1.151/B.1351.2/B.1351.3	K417N E484K N501Y D614G

The lyophilized protein was reconstructed by dissolving 100 µg of the pellet in distilled water (400 µL, pH 7.4) obtaining solutions with a concentration of 0.25 mg/ml. In our previous works (Mancini et al. 2024; D’Arco et al. 2023), other concentrations were investigated, obtaining similar results and verifying the independence of IR measurements from concentrations.

### Attenuated-total-reflection infrared spectroscopy: experimental set-up and data collection parameters

A Bruker (Billerica, MA, USA) Vertex 70v Michelson spectrometer integrated with an ATR–Diamond single reflection module and a DLaTGS wide range detector was used to collect ATR-IR spectra of monomeric S1 protein of the SARS-CoV-2 Beta variant. IR spectroscopic measurements were performed at room temperature (25 °C) and under vacuum conditions, to mitigate the interferences induced by water vapour and CO_2_ absorptions. The background spectrum of the solvent was collected before each sample measurement, with the same experimental settings as the protein solution. 5 µl of the solution (protein solution and/or water solvent) were directly dropped on the ATR diamond crystal for each ATR-IR measurement, collecting several repetitions of 128 scans between 400–4000 cm^−1^ with a spectral resolution of 2 cm^−1^. Seven independent depositions were measured and analysed. ATR crystal was cleaned with ethanol (purity > 90%), distilled water and lens tissue to eliminate any impurity. OPUS 8.2 software (Bruker Optics) and algorithms based on MATLAB (ver. 2020, MathWorks Inc., Natick, MA, USA) (Mancini et al. 2024; D’Arco et al. 2023) were used to perform the spectral analysis: absorbance calculation, baseline correction, water subtraction, ATR advanced correction, cut and average. Protein secondary structures were studied focusing on the amide I vibrational absorption band (Yang et al. 2015; Barth 2007, 2000), lying in the spectral range from 1590 to 1720 cm^−1^ and referred to the C=O stretching. For each protein, the ATR-IR spectrum of the amide I band was normalized to the maximum value and deconvoluted into its spectral components. The minima of 2nd-derivative absorption spectrum were used to obtain the spectral frequencies as starting points for multiple gaussian fittings, performed with OPUS 8.2 software (Mancini et al. 2024; D’Arco et al. 2023; Yang et al. 2015; Barth 2007, 2000), considering the residual error (RMSE) value as goodness-of-fit parameter.

The area of each convoluted band was normalized to the total integrated intensity and used to calculate the percentage contribution of each absorption band, which was then used to estimate the percentage content of protein secondary structures (Yang et al. 2015; Barth 2007; Wolpert and Hellwig 2006). The error associated with each secondary structure percentage content was calculated by propagating the standard deviations of the percentage contribution of the convoluted band integrals, obtained from each protein measurement run by adapting the final fit to its corresponding spectrum (Ricciardi et al. 2021).

### ColabFold, DSSP assignment, molecular dynamics simulation and protein-sol software

The three-dimensional (3D) structure prediction of S1 protein was computed to ColabFold software (Mirdita et al. 2022) starting from the FASTA amino acid sequence, outperforming AlphaFold2 (Jumper et al. 2021) algorithm. PyMOL (https://pymol.org/) was used to visualize the PDB file obtained from the calculations.

The obtained atomic file was then analysed with DSSP server (http://bioinformatica.isa.cnr.it/SUSAN/NAR2/dsspweb.html) for the static assignments of secondary structures, based on the analysis of backbone dihedral angles and hydrogen bonds.

ColabFold-predicted structure was used as a model for Molecular Dynamics (MD) simulations and post-processing analyses, performed with the GROMACS v. 2022.3 package (Spoel et al. 2005; Berendsen et al. 1995). The centre of mass of the protein was placed at the centre of a cubic simulation box with dimensions ensuring that adjacent images were at least 10 Å apart. The box was solvated with TIP3P water molecules and 0.15 M of NaCl was added to make the system neutral. CHARMM22* force field was employed to model the Beta variant protein for its ability to reproduce the transitions between α-helix and β-sheet secondary structures, as well as to maintain a balanced representation of α-helix and random coil conformations. The Urey-Bradley term, incorporated in the CHARMM22* force field (MacKerell et al. 1998), accounts for angle bending through 1,3 nonbonded interactions, thereby enhancing the accuracy of molecular vibration assessments compared to other commonly used force fields.

A minimization phase was performed, for 5·10^3^ + 5·10^3^ steps of steepest descent and conjugate gradient algorithms in series, with a maximum force value of 10 kJ·mol^−1^·nm^−1^. Constraints on all the hydrogen bonds were imposed with the LINCS algorithm (Hess et al. 1997) for all the simulation phases. We performed 3 × 20 ns simulations in the NVT ensemble at different temperatures (150, 200, and 300 K), applying position restraints on the protein backbone and side chains to relax the solvent around the protein, followed by a 20 ns simulation in the NPT ensemble without restraints to complete the equilibration process. After that, 600 ns production simulations were performed in the NpT ensemble. The V-rescale thermostat (Bussi et al. 2007) was used to fix the temperature of the whole system at room temperature, with a coupling time of 0.1 ps, and the Parrinello-Rahman barostat (Nosé and Klein 1983; Parrinello and RahMan 1981) was used to fix the pressure at 1 bar, with a coupling time of 2 ps and an isothermal compressibility of 4.5·10^−5^ bar^−1^. To handle the Coulomb interactions, the Particle Mesh Ewald algorithm (Darden et al. 1993) was implemented with a time step of 2 fs and a non-bonded pair list cut-off of 1.0 nm. The list was updated every 10 steps. GROMACS and Python v. 3 (Van Rossum and Drake 2009) were used to analyze the numerical data obtained from the simulations, and Visual Molecular Dynamics v. 1.9.3 tool (Humphrey et al. 1996) was used for trajectory visualization and analysis, as needed. FES heatmaps are computed in two dimensions, using the RMSD and the Rg. The free energy is defined in relation to the canonical partition function through the logarithmic term, as follows:

$$G=-0.001\bullet \mathrm{Av}\bullet \mathrm{Kb}\bullet T\bullet ({\mathrm{log}}_{10}(Z)-{\mathrm{log}}_{10}(\mathrm{max}(Z)))$$

*G* is expressed in kcal/mol, with Kb = 3.2976268E24 (cal/K) that is the Boltzmann constant, Av = 6.0221417923 is the Avogadro number, and *T* = 298 (K) the temperature. The square matrix *Z* contains the frequency of occurrence of pairs of RMSD and Rg values.

Protein-sol software (https://protein-sol.manchester.ac.uk/) was employed to compute hydrophobicity patches on the S1 protein surface (Hebditch et al. 2017), computing the non-polar to polar (NPP) ratio surface.

Expasy ProtParam web-server (https://web.expasy.org/protparam/) was used to calculate an estimation of the number, percentage and characteristic of each amino acid presents in the protein.

## Results

### Attenuated-total-reflection infrared spectroscopy

SARS-CoV-2 monomeric S1 subunit of Beta variant was modelled by the AlphaFold algorithm (Jumper et al. 2021), outperformed by ColabFold (Mirdita et al. 2022), and visualized with PyMOL (https://pymol.org/), see Fig. 1. Here, the RBD is displayed in a darker colour and the mutation sites are zoomed in and highlighted with different colours: K417N in yellow, E484K in orange, N501Y in red and D614G in pink. The amino acid sequence of the Beta variant S1 protein is provided in Supplementary Information (SI), Figure S1.

**Fig. 2:**
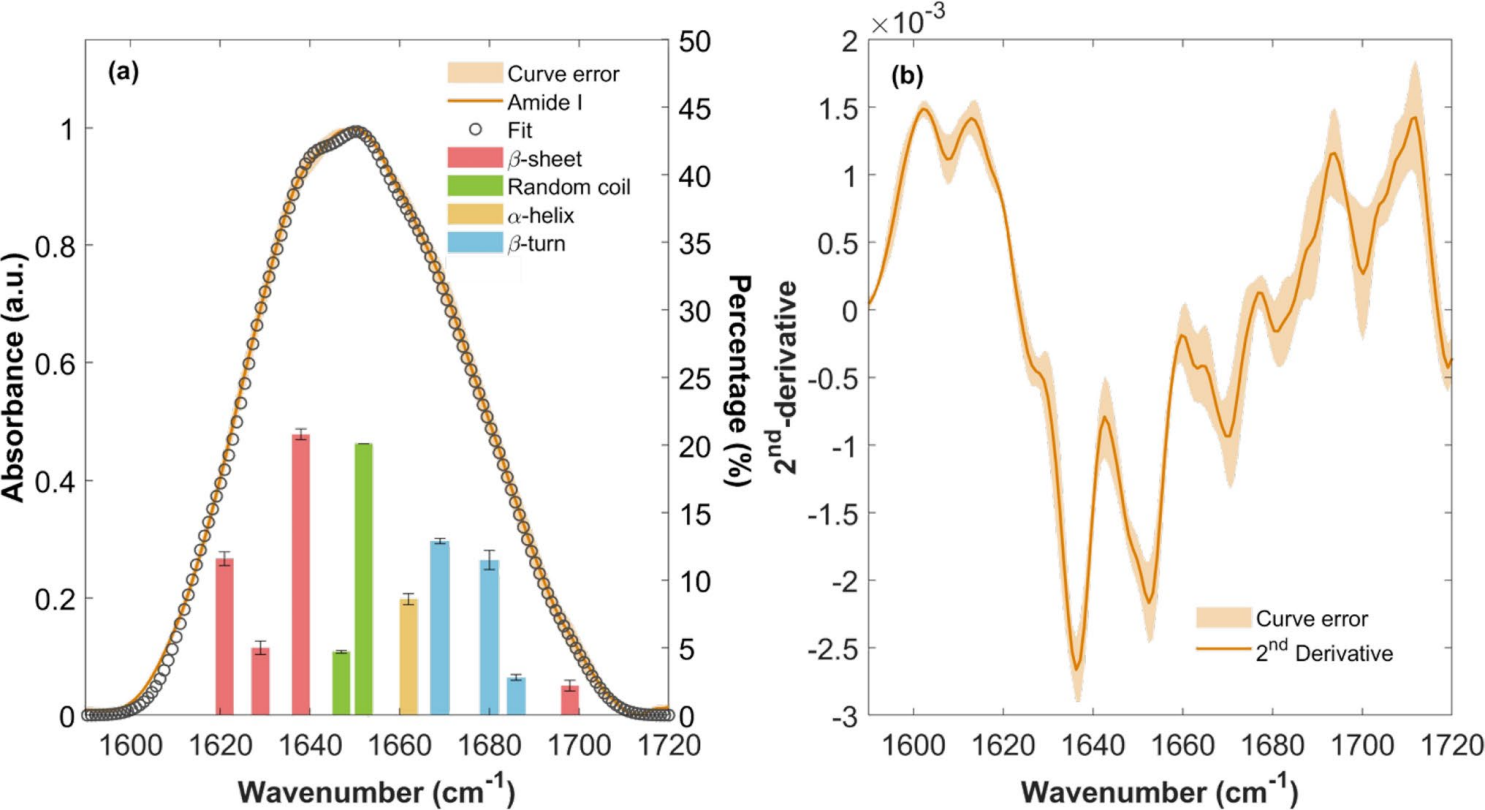
**a** Left y-axis: IR amide I band of Beta S1 protein (orange curve), its error (orange transparent area) and multi-gaussian fitting (grey circles curve). Right y-axis: percentage contribution of each spectral component (different colours). **b** Amide I second derivative (orange curve) with the curve error (orange transparent area). Data are presented as MEAN ± SD

Figure 2 displays the IR amide I absorbance A(ω) versus frequency (ω) of the SARS-CoV-2 S1 of the Beta variant (a) and the second derivative of the amide I spectrum (b), both presented as mean values within standard deviation (SD) indicated in the orange transparent area. In Fig. 2a, the IR amide I absorbance (orange line) is shown along with the total fit curve (empty grey circles), covering the range from 1590 and 1720 cm^−1^, measured at 7.4 pH and concentration of 0.25 mg ml^⁻1^. Assuming that the area of each IR spectral component is proportional to the relative amount of the corresponding secondary structure, the convoluted bands are represented by underlying bars, with their heights indicating the percentage contribution of each spectral component to the overall amide I band intensity (Yang et al. 2015, 2022; Kong and Yu 2007; GoorMaghtigh et al. 1994). The assignment to a specific secondary structure is indicated using different color codes (pink for β-sheet, green for random coil, yellow for α-helix, and blue for β-turn).

IR absorption amide I band presents the maximum located at (1650 ± 1) cm^−1^, and with a value of 56 cm^−1^ for the Full Width at Half Maximum (FWHM), in accordance with the amide I band maximum observed for the WT S1 protein (Mancini et al. 2024; D’Arco et al. 2023).

β-sheet vibrations (pink bars, in Fig. 2a) arise with two different contributions, one at low frequencies due to the ν_⟂_ mode, and the other one at high frequency attributable to ν_//_ mode (Yang et al. 2015, 2022; Barth 2007, 2000; Kong and Yu 2007): the vibrations are located at 1621, 1629, 1638 and 1698 cm^−1^ (see Table S2). The spectral component located at 1621 cm^−1^ is associated with the contribution coming from parallel β-sheet, meanwhile the vibrations at higher frequencies, 1629, 1638 and 1698 cm^−1^, are related to antiparallel β-sheet vibrations (Barth 2007, 2000; Kong and Yu 2007; Lahiri et al. 2024). The α-helix contribution (yellow bars, in Fig. 2a) has a single absorption peak located at 1662 cm^−1^. Random coil bands (green bars, in Fig. 2a) rise at 1647 and 1652 cm^−1^, while the β-turn absorption bands (blue bars in Fig. 2a) are located at 1669, 1680 and 1686 cm^−1^. However, the β-turn signature at 1686 cm^−1^ may in part also contribute to β-sheet signatures (Barth 2007, 2000; Kong and Yu 2007; Yang et al. 2022; GoorMaghtigh et al. 1994).

Table 2 summarizes the percentage content of each secondary structure, in terms of the average and SD (as explained in the Materials and Methods section).

**Table 2:** Secondary structure percentage content from the IR analysis. Percentage values were estimated from amide I band deconvolution with a multi-Gaussian fit for Beta variant monomeric S1 protein (see Fig. 1)

β-sheet (%)	α-helix (%)	Random coil (%)	β-turn (%)
39 ± 2	8 ± 1	24 ± 1	27 ± 2

Data are presented as MEAN ± SD in terms of percentage content

Similarly to the WT S1 protein(Mancini et al. 2024; D’Arco et al. 2023), overall, we observed that also the Beta variant shows the presence of highly ordered structures such as β-sheets (39%) and α-helices (~ 8%), attributable to greater mechanical stability to the structure, while unordered structures constitute the 24% of its composition.

### AlphaFold2, DSSP assignment and molecular dynamics simulations

Starting from the amino acid sequence provided by Sino Biological Europe GmbH (Eschborn, Germany), S1 protein of Beta variant was modelled by AlphaFold2 (Jumper et al. 2021) through the ColabFold interface (Mirdita et al. 2022). The algorithm predicted two distinct possible stable conformations: “open” and “closed”. In the “closed” conformation, the RBD and the N-terminal domain (NTD) are positioned very close to each other, resulting in a compact and folded conformation. In the “open” conformation, instead, the RBD and NTD domains are positioned farther apart. Based on the two conformations, we performed DSSP calculations to estimate the secondary structure percentage present in the protein. Carrying out MD simulations via GROMACS v.2022.3 package (Spoel et al. 2005; MacKerell et al. 1998) (see Materials and Methods), we predicted and simulated how the protein behaves and adapts in an aqueous environment under serological condition pH 7.4. Both DSSP assignments and MD simulations contribute to the analysis of protein secondary structure, each providing distinct and complementary information. DSSP results refer to a static model: the amino acid sequence is analyzed, and the structural assignment is based on the protein in an isolated, static conformation. In contrast, MD simulations provide insights into the dynamic behaviour of the protein over time, modelling its evolution in a simulated serological environment.

DSSP-web tool (Kabsch and Sander 1983) was employed for the secondary structure assignment starting from atomic coordinates files. In Table 3, the DSSP secondary structure assignments for S1 Beta variant protein, calculated both in “open” and “closed” conformations, are reported.

**Table 3:** Secondary structure percentage content from DSSP assignments. Errors are assumed to be 20% of the value (Kabsch and Sander 1983)

β-sheet (%)		α-helix (%)		Random coil (%)		β-turn (%)	
Open	Closed	Open	Closed	Open	Closed	Open	Closed
41 ± 8	42 ± 8	8 ± 2	8 ± 2	31 ± 6	30 ± 6	20 ± 5	20 ± 4

**Fig. 3:**
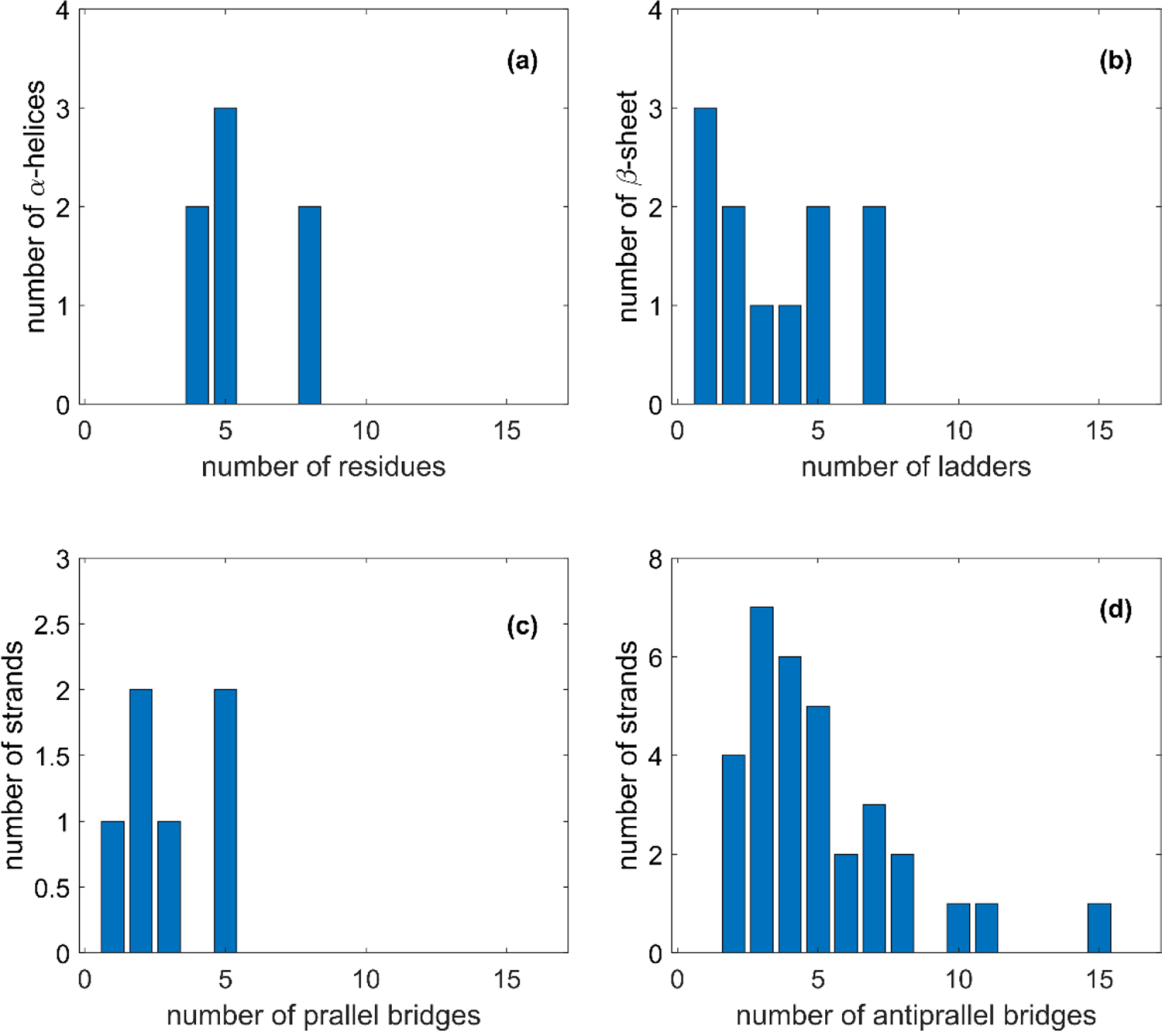
Histograms of number of **a** α‐helices having a certain number of residues, b β‐sheet structures having a certain number of ladders and strands having a certain number of **c** parallel and **d** antiparallel bridges

Figure 3 shows the frequencies of α-helices having a certain number of residues (a), the number of ladders contained in the β-sheet structures (b) and the number of strands having parallel (c) and antiparallel (d) bridges.

The results highlight that, as for the WT (Mancini et al. 2024), the S1 unit presents short α-helix structures, which contain a low number of residues. Even for the β-sheet, the number of ladders is lower than ten, indicating that the structures are formed by short domains. From the comparison between parallel and antiparallel number of bridges for strands, we can observe that mostly there are more longer strands containing antiparallel bridges with respect to the parallel ones.

**Fig. 4:**
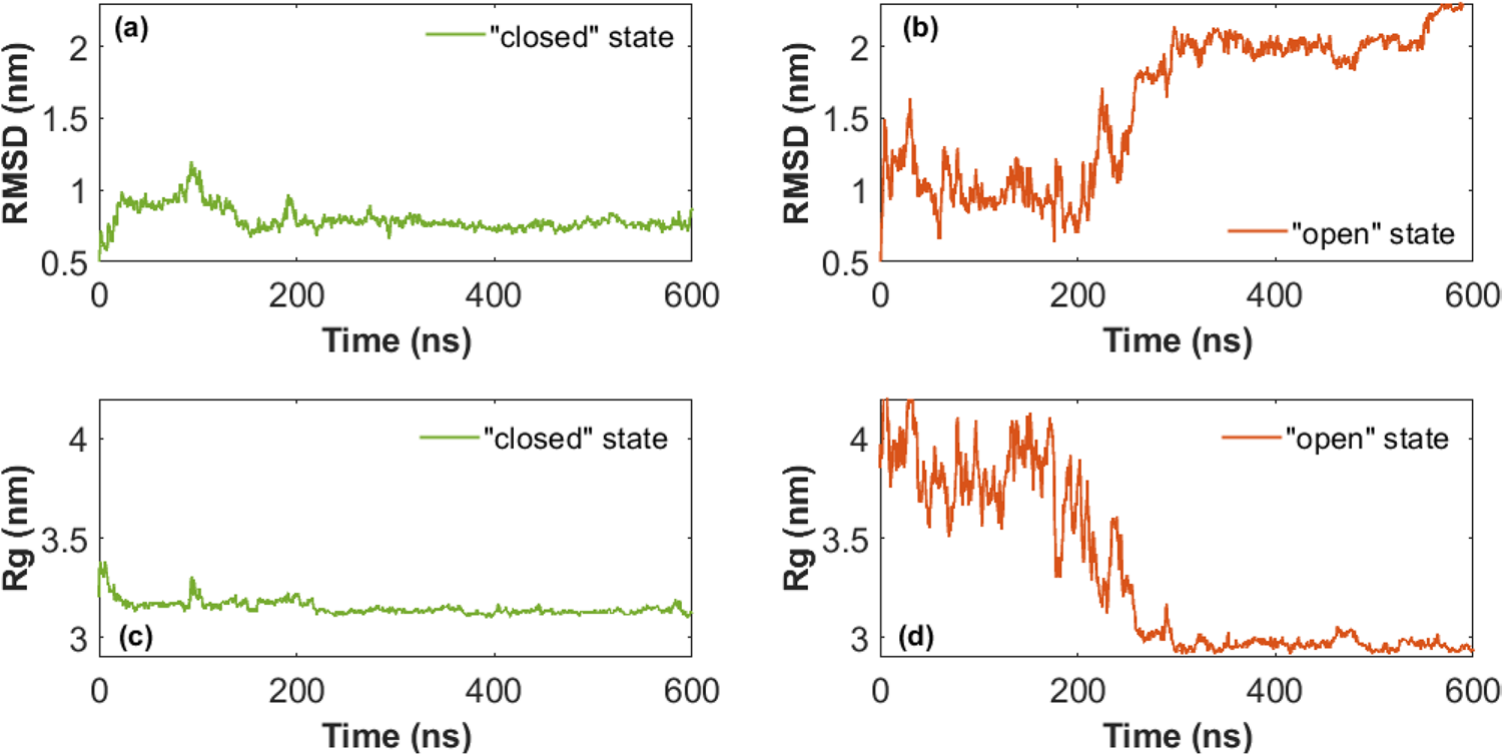
RMSD (up) and Rg (bottom) curves during 600 ns of MD simulations of S1 protein of Beta variant, starting both from the “closed” (green curve) and the “open” (orang curve) conformation: RMSD from “closed” (**a**) and “open” conformation (**b**), Rg from “closed” (**c**) and “open” conformation (**d**)

Furthermore, MD simulations were performed in both the “open” and the “closed” conformations for the S1 protein of the Beta variant. CHARMM22* force field was applied, observing the protein behaviour for 600 ns (numerical convergence timing) (Russell et al. 2015; Zhao et al. 2018). This was evaluated by observing the Root Mean Square Deviations (RMSD) values of atomic positions throughout the simulation, reported in Fig. 4. The time-dependent behaviour of the Radius of Gyration (Rg) was studied for both models and reported in Fig. 4.

In the case of the initial “open” conformation, the RMSD curve presents a drift towards bigger values. This indicates that the protein is dynamically reaching a new conformation. Meanwhile, the RMSD of the “closed” conformation preserves a quite stable value over time, showing a more stable behaviour. The same time-dependent trend is obtained observing Rg value. The closed model retains a quite stable Rg value and preserves the closed configuration along the 600 ns MD simulation. Instead, the initially open model tends to rearrange over time, decreasing the Rg value until achieving a folded and closed conformational structure around 300 ns of simulation time, showing a smaller Rg with respect also to the closed initial and final conformations.

Initial and final Rg values for both “open” and “closed” conformations were calculated as the average over the first 0–100 ns and the last 500–600 ns of simulation, respectively, and summarized in Table 4.

**Table 4:** Rg and RMSD values in nm calculated both in closed and open conformations. Rg values are obtained as the MEAN ± SD over the first and last 100 ns of MD simulations

Radius of Gyration (nm)			
Open		Closed	
Initial	Final	Initial	Final
3.81 ± 0.14	2.96 ± 0.02	3.17 ± 0.03	3.13 ± 0.02

RMSD (nm)	
Open	Closed
0.78 ± 0.05	2.15 ± 0.12

RMDS values are calculated as the average on the last 100 ns of the MD simulation of S1 proteins

Percentage contents of secondary structure as calculated from MD simulations are reported in Table 5 for both open and closed conformations.

**Table 5:** Radius of Gyration (Rg) values in nm calculated both in closed and open conformations. Values are obtained as the MEAN ± SD over the last 100 ns of simulation

β-sheet (%)		α-helix (%)		Random coil (%)		β-turn	
Open	Closed	Open	Closed	Open	Closed	Open	Closed
36 ± 4	37 ± 3	5 ± 2	6 ± 2	34 ± 3	29 ± 3	25 ± 2	28 ± 2

Free Energy Surface (FES) values have been calculated, and two-dimensional heatmaps were computed as a function of RMSD and Rg values, as reported in Figure S4, both for the systems starting from the initial “closed” (left panel) and “open” conformation (right panel). The free energy leaded the colour map and shows the possible pairs probability values for the quantities under consideration. The data are represented in the phase space [Rg; RMSD], where each point corresponds to a protein configuration recorded every 0.1 ns. In the case of the closed conformation, the diffusion area of points in phase space is smaller compared to the diffusion area for the “open” conformation. This indicates that the protein needs to explore a smaller range of Rg values to achieve equilibrium, whereas the initial “open” conformation requires spanning a larger space before finding the most stable configuration. The lowest FES value is obtained at the final Rg value, as expected, meaning that it is the most stable conformation.

MD simulations provided insights into the stability of the two AlphaFold models predicted from the amino acid sequence. Numerical analyses of the Rg and the two-dimensional heatmaps revealed that the closed conformation retains its structural integrity upon to the solvent. In contrast, the open model undergoes a conformational rearrangement, adopting a more compact and closed-like structure. Therefore, we decided to maintain the closed model as the best conformation, referring to the closed conformation for the secondary structures analysis and for the hydrophobic calculations.

### Hydrophobic calculations and surface polarity computation

To understand the 3D conformation of the protein, which is influenced by the hydropathic properties of its amino acids (Damodharan and Pattabhi 2004) and the solvent interactions, we also performed an amino acids composition analysis (see Table S3) and calculated the Grand Average of Hydropathicity (Gravy) value (Kyte and DooLittle 1982) based on the protein primary sequence. The estimation of the number and percentage of each amino acid present in the protein was obtained via the Expasy ProtParam web-server (https://web.expasy.org/protparam/) (Wilkins et al. 1999). We observed that the protein is predominantly composed of hydrophobic amino acids (around 40%), among them Valine (Val), Leucine (Leu) and Phenylalanine (Phe) are present in percentage of 7.8%, 7.3% and 6.9%, respectively. The content of polar amino acids is estimated around 27%, especially Threonine (Thr), Serine (Ser) and Asparagine (Asn), with 8.4%, 7.3% and 7.9%, respectively. Finally, the remaining 33% consists of aromatic and charged amino acids.

The Gravy value is a parameter that gives an estimation of the protein behaviour taking into account the properties of the entire amino acids sequence. In particular, a positive Gravy value is indicative of a hydrophobic protein, whereas a negative Gravy value is representative of hydrophilic protein behaviour. From its calculation for the Beta S1 protein, we obtained a value of −0.257 which is indicative of a slight global hydrophilic behaviour as the WT of SARS-CoV 2 S1 (Gravy value −0.256) (Mancini et al. 2024; Kyte and DooLittle 1982; Ma et al. 2018; Bagag et al. 2013). From the same amino acid composition, we computed the instability index values to support this observation (Guruprasad et al. 1990; GaMage et al. 2019). For the two proteins, we obtained value of 28.03 and 27.40 for Beta variant and WT, respectively. Both proteins have instability index values well below 50 (Hebditch and Warwicker 1969), meaning they are predicted to be stable under in vitro conditions, despite mutations in the Beta variant. Since the mutations are mainly located in the RBD region, the computation highlights that they affect the protein's hydrophilic behaviour without impacting its stability.

**Fig. 5:**
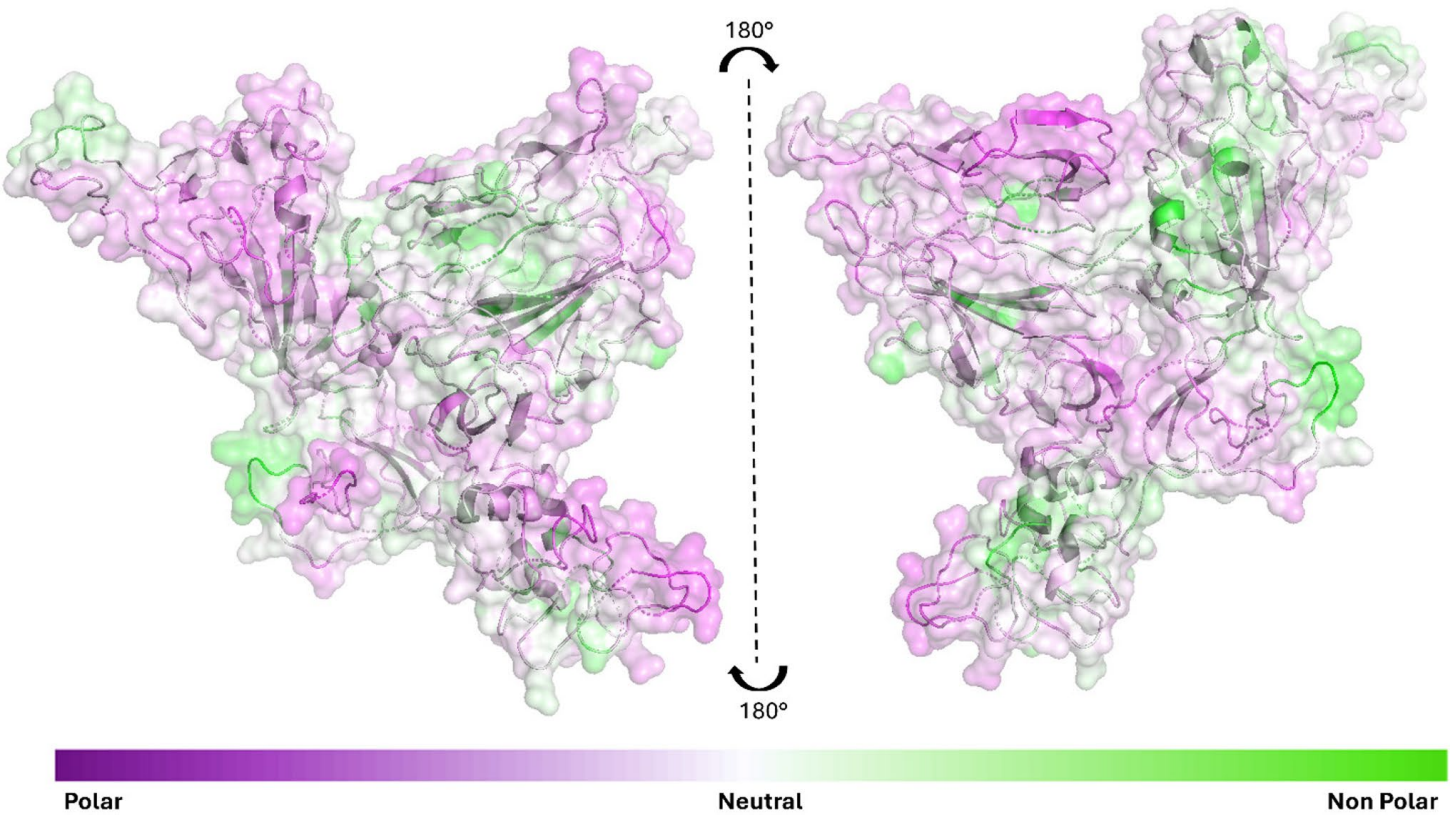
NPP surface ratio of the Beta variant S1 protein. NPP ratio distribution is computed for the S1 surface of “closed” model S1. The colour scale is shown on the bottom: purple corresponds to hydrophilic, white to neutral and green to hydrophobic behaviour

To clarify the relationship between hydrophilic properties, solvent interactions and 3D structure of the protein, we computed Non-Polar to Polar (NPP) surface ratio maps (Hebditch et al. 2017; Hebditch and Warwicker 1969), using Protein-sol software (https://protein-sol.manchester.ac.uk/), evaluating the hydrophobic clusters. NPP ratio estimates the distribution of surface polarity, as shown in Fig. 5. Figure 5 shows the protein with an emphasis on its secondary structure and surface characteristics. The surface is color-coded to indicate hydrophobicity, ranging from polar (hydrophilic) to non-polar (hydrophobic) regions. The color scale is shown at the bottom of Fig. 5, with purple representing polar regions and green representing non-polar regions.

The protein exposes its polar amino acids to the solvent, resulting in a hydrophilic surface, while the hydrophobic amino acids are compacted in the inner regions. This is particularly evident in the RBD domain, which, despite being overall hydrophilic, contains a high number of non-polar amino acids in its interior, as displayed in Figure S5.

## Discussions and conclusions

The spread of the SARS-CoV-2 pandemic and its rapid circulation have given rise to new mutated forms of the virus, which present a higher infectious capability and affinity for human receptors. In this paper, we investigated, for the first time, secondary structure of the monomeric S1 protein of the SARS-CoV-2 Beta variant through a multidisciplinary approach combining IR spectroscopy and computational methods.

IR deconvolution analysis highlights distinct shapes and distributions of amide I spectral components, corresponding to various vibrational modes of protein structures. Among them, β-sheet modes appear as four distinct components at 1621, 1629, 1638 and 1698 cm^−1^ (pink bars in Fig. 2a). The parallel β-sheet contribution is attributed to the band at 1621 cm^−1^, accounting for 11% of the total protein secondary structure. In contrast, the bands at 1629, 1638 and 1698 cm^−1^ bands are associated with antiparallel β-sheet, contributing 5%, 21% and 2%, respectively. According to literature sources (Barth 2007, 2000), antiparallel β-sheets with a high number of ladders can cause a shift in the absorbance peak toward lower frequencies (~ 1630 cm^−1^). We observed the component at 1629 cm^−1^, slightly shifted to a lower frequency compared to the expected band at 1630 cm^−1^, which may be associated with β-sheets containing a high number of ladders. In contrast, the IR peaks at 1638 cm^−1^ and 1698 cm^−1^ can be attributed to antiparallel β-sheets with a lower number of ladders.

These results and observations are in agreement with the DSSP estimations of ladders and bridges, particularly highlighting the presence of parallel and antiparallel β-strands, as shown in Fig. 3b–d. A comparison between Fig. 3c, d reveals a greater number of antiparallel β-strands than parallel ones, respectively, consistent with the spectroscopic results (28% antiparallel β-sheet vs 11% parallel β-sheet). Furthermore, Fig. 3b shows that the majority of β-sheet have short ladders (≤ 5), whereas only a few β-sheets present a large number of ladders (~ 7). DSSP calculations indicate that parallel β-sheets consist solely of short ladders, while antiparallel β-sheets exhibit a bimodal ladders distribution.

Figure 3a shows that the Beta variant S1 protein contains a total number of 7 α-helices, each composed of a relatively small number of residues (< 10). This observation is consistent with the IR data, which indicate a weak α-helix absorption peak at 1662 cm^−1^, accounting for approximately 8% of the total signal.

**Fig. 6:**
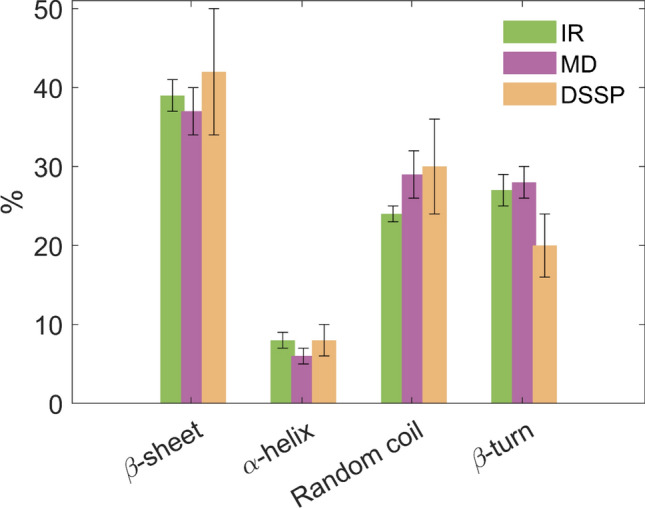
Direct comparison between the secondary structure content estimation from IR, MD and DSSP simulations. The bars are reported within the error technique and with different colours: IR in green, MD in purple and DSSP in ochre

In Fig. 6, we further provide a graphical comparison of the secondary structure obtained from the three different analyses: in green the IR estimations, in purple the MD computations and in ochre the DSSP results. The values from each technique are reported within their error bars, see Materials and Methods.

We observed that the DSSP calculations exhibit the largest deviations; however, the results remain consistent with those obtained from the other two techniques. DSSP tends to overestimate the β-sheet content while underestimating the proportion of β-turns. The IR and MD data are in agreement within the error bars, reporting the comparable values for both β-sheet and β-turn structures, and consistently identifying the β-sheet as the predominant secondary structure in the S1 Beta protein. As is well known, β-sheet structures are mechanically more stable than other secondary structure types, which may contribute to the notable stability and high adaptability of the Spike protein (Bagag et al. 2013; Upadhyay et al. 2021).

The amino acid composition analysis reveals that the Beta S1 protein is predominantly composed of hydrophobic amino acids (~ 40%), with Val, Leu, and Phe being the most abundant. Despite the high hydrophobic amino acid content, the calculated Gravy value of −0.257 indicates that the Beta S1 protein exhibits an overall slightly hydrophilic behaviour, comparable to the WT SARS-CoV-2 S1 protein (−0.256).

The NPP ratio maps show extensive hydrophilic zones distributed across the protein surface, resulting in larger hydrophilic areas (purple regions in Fig. 5) compared to the neutral and hydrophobic ones (white and green regions, respectively). This hydrophilic behaviour is particularly important, considering that this region of the protein is the first to be involved in the infection process by anchoring to the human host receptor. Indeed, the balance between hydrophilicity and hydrophobicity in the binding regions plays a crucial role in the recognition and interaction between proteins. In the RBD domain, a hydrophilic surface coexists with a core enriched in non-polar amino acids, indicating structural compactness despite surface polarity. Observing the instability index values, based on amino acid composition, they are below the threshold of 50 for both the Beta variant (28.03) and the WT (27.40), suggesting low intrinsic stability, although this is unaffected by the mutations localized in the RBD. These findings suggest that mutations in the Beta variant affect surface hydrophilicity but do not significantly alter overall structural stability or folding behaviour, as supported by MD simulations.

In conclusion, the multidisciplinary approach by combining different experimental and computational techniques, our work provides insightful information on SARS-CoV-2 Beta variant protein secondary structures, shedding light on important aspects, such as its hydrophobicity and conformational order. Understanding the secondary structural characteristics of the SARS-CoV-2 S protein and its components is of primary importance for elucidating the mechanisms involved in the viral process and for guiding targeted actions aimed at the development of specific drugs, preventive strategies, and diagnostic tools, such as the design of optical biosensors. 

## Supplementary Information

Below is the link to the electronic supplementary material.

Supplementary file1 (DOCX 452 KB)

## Data Availability

The data that support the findings of this study are available from the corresponding author upon reasonable request.

## References

[CR1] Bagag A, Jault J-M, Sidahmed-Adrar N, Réfrégiers M, Giuliani A, Le Naour F (2013) Characterization of hydrophobic peptides in the presence of detergent by photoionization mass spectrometry. PLoS ONE 8:e79033. 10.1371/journal.pone.0079033 24236085 10.1371/journal.pone.0079033 PMC3827311

[CR2] Barth A (2000) The infrared absorption of amino acid side chains. Prog Biophys Mol Biol 74:141–173. 10.1016/s0079-6107(00)00021-3 11226511 10.1016/s0079-6107(00)00021-3

[CR3] Barth A (2007) Infrared spectroscopy of proteins. Biochim Biophys Acta 1767:1073–1101. 10.1016/j.bbabio.2007.06.004 17692815 10.1016/j.bbabio.2007.06.004

[CR4] Berendsen HJC, van der Spoel D, van Drunen R (1995) GROMACS: a message-passing parallel molecular dynamics implementation. Comput Phys Commun 91:43–56. 10.1016/0010-4655(95)00042-E

[CR5] Bussi G, Donadio D, Parrinello M (2007) Canonical sampling through velocity rescaling. J Chem Phys 126:014101. 10.1063/1.2408420 17212484 10.1063/1.2408420

[CR6] Choi JY, Smith DM (2021) SARS-CoV-2 variants of concern. Yonsei Med J 62:961. 10.3349/ymj.2021.62.11.961 34672129 10.3349/ymj.2021.62.11.961 PMC8542474

[CR7] Corbett KS, Gagne M, Wagner DA, O’Connell S, Narpala SR, Flebbe DR, Andrew SF, Davis RL, Flynn B, Johnston TS et al (2021) Protection against SARS-CoV-2 beta variant in MRNA-1273 vaccine-boosted nonhuman primates. Science 374:1343–1353. 10.1126/science.abl8912 34672695 10.1126/science.abl8912

[CR8] D’Arco A, Di Fabrizio M, Mancini T, Mosetti R, Macis S, Tranfo G, Della Ventura G, Marcelli A, Petrarca M, Lupi S (2023) Secondary structures of MERS-CoV, SARS-CoV, and SARS-CoV-2 spike proteins revealed by infrared vibrational spectroscopy. Int J Mol Sci 24:9550. 10.3390/ijms24119550 37298500 10.3390/ijms24119550 PMC10253540

[CR9] Damodharan L, Pattabhi V (2004) Hydropathy analysis to correlate structure and function of proteins. Biochem Biophys Res Commun 323:996–1002. 10.1016/j.bbrc.2004.08.186 15381098 10.1016/j.bbrc.2004.08.186

[CR10] Darden T, York D, Pedersen L (1993) Particle mesh ewald: an *N* ⋅log(*N*) method for ewald sums in large systems. J Chem Phys 98:10089–10092. 10.1063/1.464397

[CR11] Davies NG, Abbott S, Barnard RC, Jarvis CI, Kucharski AJ, Munday JD, Pearson CAB, Russell TW, Tully DC, Washburne AD et al (2021) Estimated transmissibility and impact of SARS-CoV-2 lineage B117 in England. Science. 10.1126/science.abg3055 33658326 10.1126/science.abg3055 PMC8128288

[CR12] Funari R, Chu K-Y, Shen AQ (2020) Detection of antibodies against SARS-CoV-2 spike protein by gold nanospikes in an opto-microfluidic chip. Biosens Bioelectron 169:112578. 10.1016/j.bios.2020.112578 32911317 10.1016/j.bios.2020.112578 PMC7467868

[CR13] Gamage DG, Gunaratne A, Periyannan GR, Russell TG (2019) Applicability of instability index for in vitro protein stability prediction. Protein Pept Lett 26:339–347. 10.2174/0929866526666190228144219 30816075 10.2174/0929866526666190228144219

[CR14] Gamage SST, Pahattuge TN, Wijerathne H, Childers K, Vaidyanathan S, Athapattu US, Zhang L, Zhao Z, Hupert ML, Muller RM et al (2022) Microfluidic affinity selection of active SARS-CoV-2 virus particles. Sci Adv. 10.1126/sciadv.abn9665 36170362 10.1126/sciadv.abn9665 PMC9519043

[CR15] Goormaghtigh E, Cabiaux V, Ruysschaert J-M (1994) Determination of soluble and membrane protein structure by fourier transform infrared spectroscopy. Sub-Cell Biochem 23:4405–4450 10.1007/978-1-4615-1863-1_10 7855879

[CR16] Guruprasad K, Reddy BVB, Pandit MW (1990) Correlation between stability of a protein and its dipeptide composition: a novel approach for predicting in vivo stability of a protein from its primary sequence. Protein Eng des Sel 4:155–161. 10.1093/protein/4.2.155 10.1093/protein/4.2.155 2075190

[CR17] Hebditch M, Warwicker J (1969) Web-based display of protein surface and PH-dependent properties for assessing the developability of biotherapeutics. Sci Rep 2019:9. 10.1038/s41598-018-36950-8 10.1038/s41598-018-36950-8 PMC6374528 30760735

[CR18] Hebditch M, Carballo-Amador MA, Charonis S, Curtis R, Warwicker J (2017) Protein-sol: a web tool for predicting protein solubility from sequence. Bioinformatics 33:3098–3100. 10.1093/bioinformatics/btx345 28575391 10.1093/bioinformatics/btx345 PMC5870856

[CR19] Hess B, Bekker H, Berendsen HJC, Fraaije JGEM (1997) LINCS: a linear constraint solver for molecular simulations. J Comput Chem 18:1463–1472. 10.1002/(SICI)1096-987X(199709)18:12<1463::AID-JCC4>3.0.CO;2-H

[CR20] Humphrey W, Dalke A, Schulten K (1996) VMD: visual molecular dynamics. J Mol Graph 14(33–38):27–28. 10.1016/0263-7855(96)00018-5 10.1016/0263-7855(96)00018-5 8744570

[CR21] Jangra S, Ye C, Rathnasinghe R, Stadlbauer D, Krammer F, Simon V, Martinez-Sobrido L, García-Sastre A, Schotsaert M (2021) SARS-CoV-2 spike E484K mutation reduces antibody neutralisation. Lancet Microbe 2:e283–e284. 10.1016/S2666-5247(21)00068-9 33846703 10.1016/S2666-5247(21)00068-9 PMC8026167

[CR22] Jumper J, Evans R, Pritzel A, Green T, Figurnov M, Ronneberger O, Tunyasuvunakool K, Bates R, Žídek A, Potapenko A et al (2021) Highly accurate protein structure prediction with AlphaFold. Nature 596:583–589. 10.1038/s41586-021-03819-2 34265844 10.1038/s41586-021-03819-2 PMC8371605

[CR23] Kabsch W, Sander C (1983) Dictionary of protein secondary structure: pattern recognition of hydrogen-bonded and geometrical features. Biopolymers 22:2577–2637. 10.1002/bip.360221211 6667333 10.1002/bip.360221211

[CR24] Khan A, Zia T, Suleman M, Khan T, Ali SS, Abbasi AA, Mohammad A, Wei D-Q (2021) Higher infectivity of the SARS-CoV-2 new variants is associated with K417N/T, E484K, and N501Y mutants: an insight from structural data. J Cell Physiol 236:7045–7057. 10.1002/jcp.30367 33755190 10.1002/jcp.30367 PMC8251074

[CR25] Kitane DL, Loukman S, Marchoudi N, Fernandez-Galiana A, El Ansari FZ, Jouali F, Badir J, Gala J-L, Bertsimas D, Azami N et al (2021) A simple and fast spectroscopy-based technique for Covid-19 diagnosis. Sci Rep 11:16740. 10.1038/s41598-021-95568-5 34408169 10.1038/s41598-021-95568-5 PMC8373901

[CR26] Kong J, Yu S (2007) Fourier transform infrared spectroscopic analysis of protein secondary structures. Acta Biochim Biophys Sin (Shanghai) 39:549–559. 10.1111/j.1745-7270.2007.00320.x 17687489 10.1111/j.1745-7270.2007.00320.x

[CR27] Krammer F (2020) SARS-CoV-2 vaccines in development. Nature 586:516–527. 10.1038/s41586-020-2798-3 32967006 10.1038/s41586-020-2798-3

[CR28] Kumar N, Shetti NP, Jagannath S, Aminabhavi TM (2022) Electrochemical sensors for the detection of SARS-CoV-2 virus. Chem Eng J 430:132966. 10.1016/j.cej.2021.132966 34690533 10.1016/j.cej.2021.132966 PMC8525496

[CR29] Kyte J, Doolittle RF (1982) A simple method for displaying the hydropathic character of a protein. J Mol Biol 157:105–132. 10.1016/0022-2836(82)90515-0 7108955 10.1016/0022-2836(82)90515-0

[CR30] Lahiri P, Das S, Thakur S, Mehra R, Ranjan P, Wig N, Dar L, Bhattacharyya TK, Sengupta S, Lahiri B (2024) Fast viral diagnostics: FTIR-based identification, strain-typing, and structural characterization of SARS-CoV-2. Anal Chem 96:14749–14758. 10.1021/acs.analchem.4c01260 39215696 10.1021/acs.analchem.4c01260

[CR31] Lee JH, Kim JW, Lee HE, Song JY, Cho AH, Hwang JH, Heo K, Lee S (2024) A dual-targeting approach using a human bispecific antibody against the receptor-binding domain of the middle east respiratory syndrome coronavirus. Virus Res 345:199383. 10.1016/j.virusres.2024.199383 38697296 10.1016/j.virusres.2024.199383 PMC11074968

[CR32] Li Q, Guan X, Wu P, Wang X, Zhou L, Tong Y, Ren R, Leung KSM, Lau EHY, Wong JY et al (2020) Early transmission dynamics in wuhan, china, of novel coronavirus-infected pneumonia. N Engl J Med 382:1199–1207. 10.1056/NEJMoa2001316 31995857 10.1056/NEJMoa2001316 PMC7121484

[CR33] Ma C, Yuan C, Cao P (2018) A facile method to prepare a hydrophilic/hydrophobic metal surface by peptide. Materials 11:1289. 10.3390/ma11081289 30046023 10.3390/ma11081289 PMC6117720

[CR34] MacKerell AD, Bashford D, Bellott M, Dunbrack RL, Evanseck JD, Field MJ, Fischer S, Gao J, Guo H, Ha S et al (1998) All-atom empirical potential for molecular modeling and dynamics studies of proteins. J Phys Chem B 102:3586–3616. 10.1021/jp973084f 24889800 10.1021/jp973084f

[CR35] Manas ES, Getahun Z, Wright WW, DeGrado WF, Vanderkooi JM (2000) Infrared spectra of amide groups in α-helical proteins: evidence for hydrogen bonding between helices and water. J Am Chem Soc 122:9883–9890. 10.1021/ja001782z

[CR36] Mancini T, Macis S, Mosetti R, Luchetti N, Minicozzi V, Notargiacomo A, Pea M, Marcelli A, Ventura G, Lupi S et al (2024) Infrared spectroscopy of SARS-CoV-2 viral protein: from receptor binding domain to spike protein. Adv Sci. 10.1002/advs.202400823 10.1002/advs.202400823 PMC11497030 39001588

[CR37] Mirdita M, Schütze K, Moriwaki Y, Heo L, Ovchinnikov S, Steinegger M (2022) ColabFold: making protein folding accessible to all. Nat Methods 19:679–682. 10.1038/s41592-022-01488-1 35637307 10.1038/s41592-022-01488-1 PMC9184281

[CR38] Moran SD, Zanni MT (2014) How to get insight into amyloid structure and formation from infrared spectroscopy. J Phys Chem Lett 5:1984–1993. 10.1021/jz500794d 24932380 10.1021/jz500794d PMC4051309

[CR39] Myshakina NS, Ahmed Z, Asher SA (2008) Dependence of amide vibrations on hydrogen bonding. J Phys Chem B 112:11873–11877. 10.1021/jp8057355 18754632 10.1021/jp8057355 PMC2633779

[CR40] Nosé S, Klein ML (1983) Constant pressure molecular dynamics for molecular systems. Mol Phys 50:1055–1076. 10.1080/00268978300102851

[CR41] Parrinello M, Rahman A (1981) Polymorphic transitions in single crystals: a new molecular dynamics method. J Appl Phys 52:7182–7190. 10.1063/1.328693

[CR42] Ramanathan M, Ferguson ID, Miao W, Khavari PA (2021) SARS-CoV-2 B.1.1.7 and B1.351 spike variants bind human ACE2 with increased affinity. Lancet Infect Dis 21:1070. 10.1016/S1473-3099(21)00262-0 34022142 10.1016/S1473-3099(21)00262-0 PMC8133765

[CR43] Ricciardi V, Portaccio M, Perna G, Lasalvia M, Capozzi V, Cammarata FP, Pisciotta P, Petringa G, Delfino I, Manti L et al (2021) FT-IR transflection micro-spectroscopy study on normal human breast cells after exposure to a proton beam. Appl Sci 11:540. 10.3390/app11020540

[CR44] Russell BA, Kubiak-Ossowska K, Mulheran PA, Birch DJS, Chen Y (2015) Locating the nucleation sites for protein encapsulated gold nanoclusters: a molecular dynamics and fluorescence study. Phys Chem Chem Phys 17:21935–21941. 10.1039/C5CP02380G 26234926 10.1039/c5cp02380g

[CR45] Santos MCD, Morais CLM, Lima KMG (2021) ATR-FTIR spectroscopy for virus identification: a powerful alternative. Biomed Spectrosc Imaging 9:103–118. 10.3233/BSI-200203

[CR46] Starr TN, Greaney AJ, Hilton SK, Ellis D, Crawford KHD, Dingens AS, Navarro MJ, Bowen JE, Tortorici MA, Walls AC et al (2020) Deep mutational scanning of SARS-CoV-2 receptor binding domain reveals constraints on folding and ACE2 binding. Cell 182:1295-1310.e20. 10.1016/j.cell.2020.08.012 32841599 10.1016/j.cell.2020.08.012 PMC7418704

[CR47] Tan B, Zhang X, Ansari A, Jadhav P, Tan H, Li K, Chopra A, Ford A, Chi X, Ruiz FX et al (2024) Design of a SARS-CoV-2 papain-like protease inhibitor with antiviral efficacy in a mouse model. Science 383:1434–1440. 10.1126/science.adm9724 38547259 10.1126/science.adm9724 PMC12178660

[CR48] Tang JW, Toovey OTR, Harvey KN, Hui DSC (2021) Introduction of the South African SARS-CoV-2 variant 501Y.V2 into the UK. J Infect 82:e8–e10. 10.1016/j.jinf.2021.01.007 33472093 10.1016/j.jinf.2021.01.007 PMC7813514

[CR49] Tegally H, Wilkinson E, Giovanetti M, Iranzadeh A, Fonseca V, Giandhari J, Doolabh D, Pillay S, San EJ, Msomi N et al (2021) Detection of a SARS-CoV-2 variant of concern in South Africa. Nature 592:438–443. 10.1038/s41586-021-03402-9 33690265 10.1038/s41586-021-03402-9

[CR50] Upadhyay V, Lucas A, Panja S, Miyauchi R, Mallela KMG (2021) Receptor binding, immune escape, and protein stability direct the natural selection of SARS-CoV-2 variants. J Biol Chem 297:101208. 10.1016/j.jbc.2021.101208 34543625 10.1016/j.jbc.2021.101208 PMC8445900

[CR51] Van Der Spoel D, Lindahl E, Hess B, Groenhof G, Mark AE, Berendsen HJC (2005) GROMACS: fast, flexible, and free. J Comput Chem 26:1701–1718. 10.1002/jcc.20291 16211538 10.1002/jcc.20291

[CR52] Van Rossum G, Drake F (2009) Python 3 reference manual. CreateSpace, Scotts Valley

[CR53] Verma J, Subbarao N (2021) A comparative study of human betacoronavirus spike proteins: structure. Function and Therapeutics Arch Virol. 10.1007/s00705-021-04961-y 33483791 10.1007/s00705-021-04961-y PMC7821988

[CR54] Vosough F, Barth A (2021) Characterization of homogeneous and heterogeneous amyloid-Β42 oligomer preparations with biochemical methods and infrared spectroscopy reveals a correlation between infrared spectrum and oligomer size. ACS Chem Neurosci 12:473–488. 10.1021/acschemneuro.0c00642 33455165 10.1021/acschemneuro.0c00642 PMC8023574

[CR55] Wilkins MR, Gasteiger E, Bairoch A, Sanchez J-C, Williams KL, Appel RD, Hochstrasser DF (1999) Protein identification and analysis tools in the ExPASy server. In: Link AJ (ed) 2-D proteome analysis protocols. Humana Press, New Jersey, pp 531–552 10.1385/1-59259-584-7:531 10027275

[CR56] Wolpert M, Hellwig P (2006) Infrared spectra and molar absorption coefficients of the 20 alpha amino acids in aqueous solutions in the spectral range from 1800 to 500 cm^−1^. Spectrochim Acta A Mol Biomol Spectrosc 64:987–1001. 10.1016/j.saa.2005.08.025 16458063 10.1016/j.saa.2005.08.025

[CR57] World Health Organization Data (2025).

[CR58] Woutersen S, Hamm P (2001) Time-resolved two-dimensional vibrational spectroscopy of a short α-helix in water. J Chem Phys 115:7737–7743. 10.1063/1.1407842

[CR59] Yang H, Yang S, Kong J, Dong A, Yu S (2015) Obtaining information about protein secondary structures in aqueous solution using Fourier transform IR spectroscopy. Nat Protoc 10:382–396. 10.1038/nprot.2015.024 25654756 10.1038/nprot.2015.024

[CR60] Yang S, Zhang Q, Yang H, Shi H, Dong A, Wang L, Yu S (2022) Progress in infrared spectroscopy as an efficient tool for predicting protein secondary structure. Int J Biol Macromol 206:175–187. 10.1016/j.ijbiomac.2022.02.104 35217087 10.1016/j.ijbiomac.2022.02.104

[CR61] Zhao L, Cao Z, Bian Y, Hu G, Wang J, Zhou Y (2018) Molecular dynamics simulations of human antimicrobial peptide LL-37 in model POPC and POPG lipid bilayers. Int J Mol Sci 19:1186. 10.3390/ijms19041186 29652823 10.3390/ijms19041186 PMC5979298

