# Supplementary material for: Spectroscopic secondary structure fingerprint of β-variant of SARS-CoV-2 spike glycoprotein
Source: Eur Biophys J. 2025 Jul 21;55(2):217–28. doi: 10.1007/s00249-025-01782-8 (PMC13109278; doi:10.1007/s00249-025-01782-8)
Supplement: Supplementary file 1 — Supplementary file1 (DOCX 452 KB) [file 249_2025_1782_MOESM1_ESM.docx]

**S1. Amico acids sequence**

*Beta variant S1 protein sequence*

VNLTTRTQLPPAYTNSFTRGVYYPDKVFRSSVLHSTQDLFLPFFSNVTWFHAIHVSGTNGTKRFDNPVLPFNDGVYFASTEKSNIIRGWIFGTTLDSKTQSLLIVNNATNVVIKVCEFQFCNDPFLGVYYHKNNKSWMESEFRVYSSANNCTFEYVSQPFLMDLEGKQGNFKNLREFVFKNIDGYFKIYSKHTPINLVRDLPQGFSALEPLVDLPIGINITRFQTLLALHRSYLTPGDSSSGWTAGAAAYYVGYLQPRTFLLKYNENGTITDAVDCALDPLSETKCTLKSFTVEKGIYQTSNFRVQPTESIVRFPNITNLCPFGEVFNATRFASVYAWNRKRISNCVADYSVLYNSASFSTFKCYGVSPTKLNDLCFTNVYADSFVIRGDEVRQIAPGQTGNIADYNYKLPDDFTGCVIAWNSNNLDSKVGGNYNYLYRLFRKSNLKPFERDISTEIYQAGSTPCNGVKGFNCYFPLQSYGFQPTYGVGYQPYRVVVLSFELLHAPATVCGPKKSTNLVKNKCVNFNFNGLTGTGVLTESNKKFLPFQQFGRDIADTTDAVRDPQTLEILDITPCSFGGVSVITPGTNTSNQVAVLYQGVNCTEVPVAIHADQLTPTWRVYSTGSNVFQTRAGCLIGAEHVNNSYECDIPIGAGICASYQTQTNSPRRARAHHHHHHHHHH

**S1.** Beta variant amino acids sequence. In blue is reported the RBD region.

**S2. Table of the secondary structure contents vs frequencies**

| Frequency (cm-1) | Integrated Intensity (%) | Assignment |
| --- | --- | --- |
| 1621 | 11.6 | β-sheet |
| 1629 | 5.0 | β -sheet |
| 1638 | 20.8 | β -sheet |
| 1647 | 4.6 | Random coil |
| 1652 | 20.1 | Random coil |
| 1662 | 8.6 | α-helix |
| 1669 | 12.9 | β -turn |
| 1680 | 11.5 | β -turn |
| 1686 | 2.8 | β -turn |
| 1698 | 2.1 | β-sheet |

**Table S2.** Amide I absorption peaks of S1 proteins from the Beta variant. Secondary structure assignment derived from the Gaussian decomposition of the vibrational absorption spectra.

**S3. Table of amino acids contents**

| Amino Acid | Number | Percentage (%) | Behaviour |
| --- | --- | --- | --- |
| Alanine (Ala, A) | 36 | 5.3 | Hydrophobic |
| Arginine (Arg, R) | 29 | 4.3 | Positive charged |
| Asparagine (Asn, N) | 54 | 7.9 | Polar |
| Aspartic Acid (Asp, D) | 30 | 4.4 | Negative charged |
| Cysteine (Cys, C) | 19 | 2.8 | Hydrophobic |
| Glutamine (Gln, Q) | 26 | 3.8 | Polar |
| Glutamic Acid (Glu, E) | 22 | 3.2 | Negative charged |
| Glycine (Gly, G) | 46 | 6.8 | Hydrophobic |
| Histidine (His, H) | 19 | 2.8 | Charged |
| Isoleucine (Ile, I) | 32 | 4.7 | Hydrophobic |
| Leucine (Leu, L) | 50 | 7.3 | Hydrophobic |
| Lysine (Lys, K) | 30 | 4.4 | Positive charged |
| Methionine (Met, M) | 2 | 0.3 | Hydrophobic |
| Phenylalanine (Phe, F) | 47 | 6.9 | Aromatic |
| Proline (Pro, P) | 36 | 5.3 | Hydrophobic |
| Serine (Ser, S) | 50 | 7.3 | Polar |
| Threonine (Thr, T) | 57 | 8.4 | Polar |
| Tryptophan (Trp, W) | 7 | 1.0 | Aromatic |
| Tyrosine (Tyr, Y) | 36 | 5.3 | Aromatic |
| Valina (Val, V) | 53 | 7.8 | Hydrophobic |

**Table S3.** List of amino acids present in the protein. Number and percentage of each amino acid in the protein.

**S4. Beta variant FES maps**


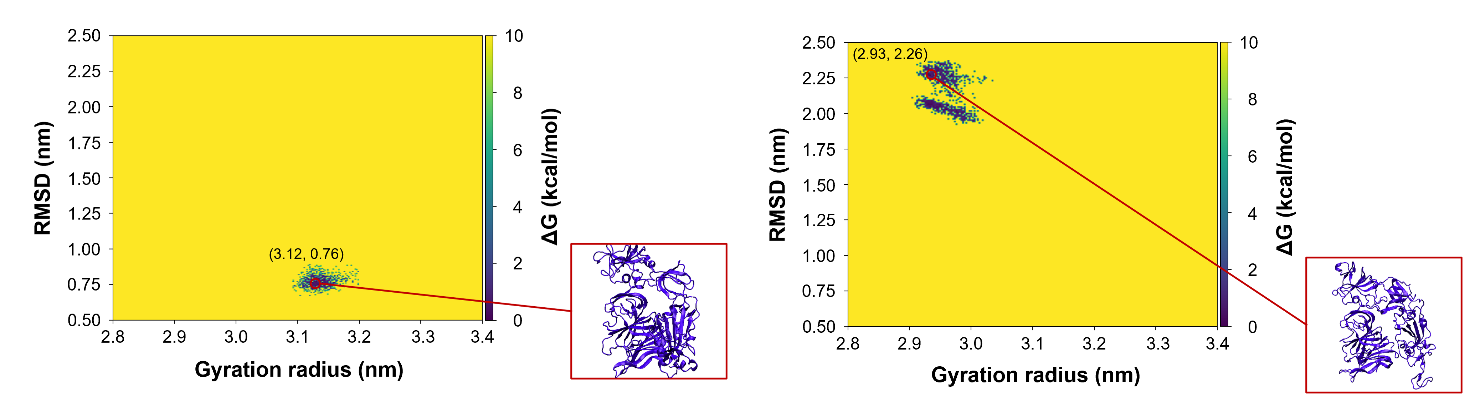


**Figure S4.** FES maps of Beta variant is computed over the entire MD simulations (600 ns), both for the systems starting from the initial “closed” state (left panel) and from the “open” state (right panel). The data are represented in the phase [Rg; RMSD], where each point represents the protein configuration recorded with a t=0.1 ns.

**S5. Beta variant hydrophobicity and hydrophilicity**


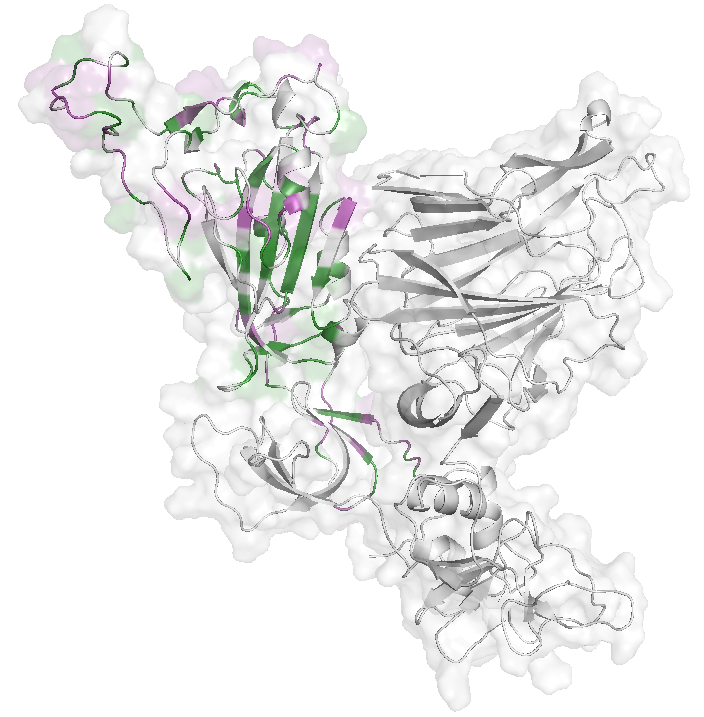


**Figure S5.** 3D Visualization of the hydrophobic and hydrophilic amino acids of the RBD of Beta Variant S1 protein. Green and purple zones are hydrophobic and hydrophilic amino acids, respectively
